# Correlated Brain Indexes of Semantic Prediction and Prediction Error: Brain Localization and Category Specificity

**DOI:** 10.1093/cercor/bhaa308

**Published:** 2020-10-27

**Authors:** Luigi Grisoni, Rosario Tomasello, Friedemann Pulvermüller

**Affiliations:** Freie Universität Berlin, Brain Language Laboratory, Department of Philosophy and Humanities, 14195 Berlin, Germany; Cluster of Excellence ‘Matters of Activity. Image Space Material’, Humboldt Universität zu Berlin, 10099 Berlin, Germany; Freie Universität Berlin, Brain Language Laboratory, Department of Philosophy and Humanities, 14195 Berlin, Germany; Berlin School of Mind and Brain, Humboldt Universität zu Berlin, 10117 Berlin, Germany; Cluster of Excellence ‘Matters of Activity. Image Space Material’, Humboldt Universität zu Berlin, 10099 Berlin, Germany; Freie Universität Berlin, Brain Language Laboratory, Department of Philosophy and Humanities, 14195 Berlin, Germany; Berlin School of Mind and Brain, Humboldt Universität zu Berlin, 10117 Berlin, Germany; Cluster of Excellence ‘Matters of Activity. Image Space Material’, Humboldt Universität zu Berlin, 10099 Berlin, Germany; Einstein Center for Neurosciences, 10117 Berlin, Germany

**Keywords:** N400, predictive coding, semantic prediction potential (SPP), semantic processing

## Abstract

With strong and valid predictions, grasping a message is easy, whereas more demanding processing is required in the absence of robust expectations. We here demonstrate that brain correlates of the interplay between prediction and perception mechanisms in the understanding of meaningful sentences. Sentence fragments that strongly predict subsequent words induced anticipatory brain activity preceding the expected words; this potential was absent if context did not strongly predict subsequent words. Subjective reports of certainty about upcoming words and objective corpus-based measures correlated with the size of the anticipatory signal, thus establishing its status as a semantic prediction potential (SPP). Crucially, there was an inverse correlation between the SPP and the N400 brain response. The main cortical generators of SPP and N400 were found in inferior prefrontal cortex and posterior temporal cortex, respectively. Interestingly, sentence meaning was reflected by both measures, with additional category-specific sources of SPPs and N400s falling into parieto-temporo-occipital (visual) and frontocentral (sensorimotor) areas for animal- and tool-related words, respectively. These results show that the well-known brain index of semantic comprehension, N400, has an antecedent with different brain localization but similar semantic discriminatory function. We discuss whether N400 dynamics may causally depend on mechanisms underlying SPP size and sources.

## Introduction

Perceiving the world is not just mapping the sensory input. When looking around or when encountering a meaningful sentence, we typically use what we have perceived to guide and predict subsequent observations. In language comprehension, predictions about subsequent phonemes, words, and even communicative actions may play an important role for the high speed with which we are able to understand (MacGregor et al. 2012; Shtyrov et al. 2014). However, some contexts do not give rise to strong predictions and, therefore, in such contexts, the unexpected stimulus will convey new, previously unpredicted information (Enns and Lleras 2008). Notably, the first-discovered and most well-known neurophysiological index of language understanding, the N400, is a brain index strongly affected by expectations, as it increases in size depending on how unexpected a critical word is within its sentence context (Kutas and Hillyard 1984; Kutas and Federmeier 2011).

Modern theories of perception and cognition put a great emphasis on the role of “prediction” and “prediction errors” (Schultz and Dickinson 2000; Friston 2010), the error arising when a prediction is falsified. For assessing and testing these theories, it is of utmost importance to have available a brain indicator of both prediction and prediction error. The N400 has been proposed to reflect prediction errors (Rabovsky and McRae 2014), although alternative interpretations exist (Pickering and Gambi 2018). A direct measure of prediction was unavailable until recently, when a brain potential building up in highly predictable contexts was discovered (Grisoni et al. 2016; Grisoni et al. 2017; León-Cabrera et al. 2017; Pulvermüller and Grisoni 2020). In some of these studies, sentence fragments such as “I take the ice cream and I …” were presented and a fast-rising negative going potential was found, which preceded the presentation of the expected sentence continuation “…lick it.” Sentences without predictable continuation did not give rise to this “semantic prediction potential,” or SPP, and, crucially, dependent on the meaning of the predicted word, different brain activation topographies and different SPP cortical sources were found. In particular, for words used to speak about mouth actions, such as “lick,” prediction-related activation was present in inferior sensorimotor cortex, where face muscles are represented, whereas sentence fragments predicting hand-related action words (e.g., “I take the pen and I … write a letter”) led to an anticipatory potential with sources in lateral sensorimotor cortex controlling hand actions (Grisoni et al. 2017). The fact that aspects of the meaning of the predictable word were revealed by the anticipatory potential supports the SPP’s role as an index of semantic predictions.

However, previous studies of semantic predictions probed very specific types of semantic predictions, involving critical words referring to body part-related actions, thus leaving the possibility that either the potential’s localization or even the results generally are specific to this subset of sentences and lexical items along with their action-related meaning. Predictions about other semantic types could either not be reflected by this neurophysiological dependent measure at all, which would question its status as a general index of prediction, or the prediction-related activity could emerge from entirely different parts of the brain (Grisoni et al. 2019).

One crucial implication of predictive coding approaches is that, the stronger a prediction about future input is, the smaller the prediction error signal will be when the expected item indeed appears. We note that this prediction is only valid if the expected item indeed appears, but not for the rare cases, where highly predictive sentence fragments are completed by an entirely unexpected item, as in the typical “N400 violations,” for example, “Joe drinks his tea with … socks.” In contrast, a lack of predictability (or a reduction of the “precision” of the prediction) implies uncertainty about the future input and therefore greater surprise and necessity to process novel, unexpected, information (Friston 2005). Although one may argue that it is trivial that a previously predicted and thus highly expected item brings about no (or little) surprise, whereas an unexpected item does, the brain correlates of this prediction–resolution (or prediction–integration) interplay are still unknown. Thus, a crucial predicted effect is an inverse correlation between the brain potential reflecting the level of “predictive semantic constraint” of a sentence context and the brain index reflecting the “semantic” prediction error immanent to the unexpected critical word. In brief, predictive coding implies that, in natural language use, an inverse correlation exists between the SPP preceding the critical word and the N400 following it. We note again that these considerations apply to common sentences as they frequently appear in natural language use. Exceptional “semantic violations” of highly predictable sentence contexts would clearly violate this rule.

**Figure 1:**
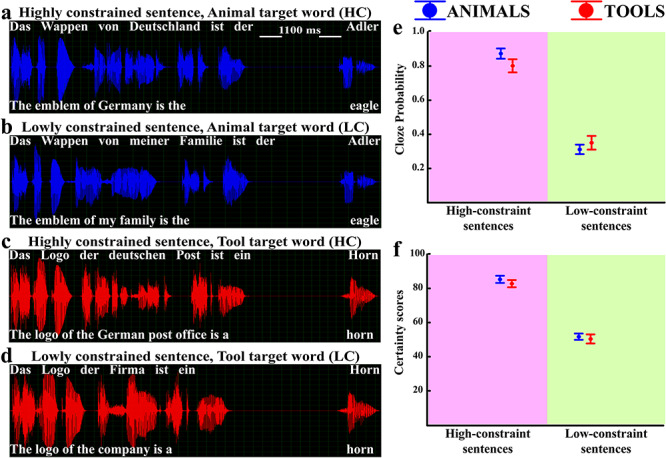
Example sentences and cloze probability and certainty scores of all the sentences used in the EEG study. At the left example sentences: (*a* and *c*) examples of HC sentences constraining the expectation of sentence-final animal (*a*) and tool (*c*) words. (*b* and *d*) Examples of LC sentences that do not strongly constrain expectations of the final word. (*e*) Cloze probability judgments of the experimental sentences are shown (means and standard errors of the mean). (*f*) “Certainty scores” of the experimental sentences are shown (means and standard errors of the mean).

To test this main prediction, we generated sentence fragments that either highly constrained the subsequent word or left it more open, which lexical items would complete the fragments. We call these the “high-” and “low-constraint conditions” (HC, LC, see Fig. 1). Since our study targets semantic brain processes in understanding sentence meaning, we used sentences with different meanings so as to obtain brain signatures of specific semantic predictions and meaning-specific prediction errors (see Fig. 1*a*–*d*). To this end, we used two well-established semantic categories, animals and tools, and created matched HC and LC sentences with identical critical words from these two categories. Previous research has shown that these categories elicit semantically specific activity in different areas of cortex, with animal nouns activating part of posterior cortical areas (i.e., inferior temporal, parietal, and occipital), possibly due to the processing of visual object knowledge about their referents, and tool nouns sparking posterior superior temporal and frontoparietal sensorimotor cortex, which may in part reflect knowledge access about the use of tools (Martin et al. 1996; Kiefer 2001; Martin 2007; Carota et al. 2012; Kiefer and Pulvermüller 2012; Carota et al. 2017; Tomasello et al. 2017). Although the SPP has previously been found to have its main sources in prefrontal cortex (Grisoni et al. 2017), we expected a degree of specificity for both semantic types, with animal sentences and words yielding specific activity indicating semantic prediction (HC) and prediction error (LC) in posterior brain areas and tool items relatively stronger activity in frontoparietal areas.

Finally, predictive coding accounts typically consider prediction and prediction error as being functionally detached along the cortical hierarchy, so that each level in the hierarchical network attempts to predict the activity at lower levels (Rao and Ballard 1999). In this framework, prediction error responses are usually thought to originate from the interaction of hierarchically organized cortical areas. Accordingly, each level of the hierarchy is engaged in predicting the pattern of activity of the next level below and prediction error responses emerge at that relatively lower level when the relatively higher one fails to make a correct prediction (Garrido, Kilner, Stephan, et al. 2009). Notably, the frontoparietal, executive/motor, and posterior perceptual/visual brain areas (Fuster 2009) are hierarchically organized so that, as we move rostrally from the primary motor (Badre and D'Esposito 2009) and anteriorly from primary visual (Van Essen and Maunsell 1983) areas, the unimodal and multimodal association territories contain neurons whose responses become progressively more sensible to complex, associative, features (i.e., higher in the hierarchy). Therefore, a crucial expectation, suggested by prediction error accounts, was to observe dissociations of prediction and prediction error responses along this functional hierarchy, with SPP sources (HC contexts) in relatively higher modality preferential brain areas and N400’s sources (LC contexts) in lower motor and visual areas.

## Materials and Methods

### Participants

Thirty healthy adults participated in this study. Participants were monolingual German native speakers with normal hearing, normal or corrected-to-normal visual acuity, and motor control. None of the participants had a record of neurological or psychiatric disease. Datasets from six participants were excluded because too many trials rejected (i.e., >30%) or because excessive alpha noise. Therefore, electrophysiological (EEG) data from 24 participants (mean age 25.5 years, ±4.9 SD; 17 females), all of them right handed, as determined by the Edinburgh Handedness Inventory (Oldfield 1971) (mean laterality quotient 77.5 ± 20.8 SD), were included in the final analysis. All participants who took part in this study provided written informed consent. Procedures were approved by the Ethics Committee of Charité Universitätsmedizin, Campus Benjamin Franklin, Berlin, Germany. Furthermore, 13 participants (mean ± SD age, 24.5 ± 3. 9 years; 8 female), all of them right handed (mean laterality quotient 81.9 ± 17.9 SD), who did not take part in the EEG experiment, were asked to listen to all sentence fragments (presented without the final “critical” noun) and list the words they expect to directly follow the fragments.

### Stimuli and Experimental Design

We created sentences about animals and tools each including a critical noun toward its end, which could easily be predicted based on the preceding fragment (e.g., translated from German: “The emblem of Germany is the eagle”). For each of these “highly constrained (HC)” sentences, a matched “low-constraint (LC)” control sentence was created that included the same critical word. For the LC sentences, it was more difficult to predict critical words from the context of the preceding sentence fragments, as these fragments invited a range of possible continuations. This resulted in 4 sentence categories (examples are sentences from the experiment translated from German into English):

HC animal: “The emblem of Germany is the eagle.”

LC animal: “The emblem of my family is the eagle.”

HC tool: “The logo of the German post office is a horn.”

LC tool: “The logo of the company is a horn.”

Figure 1*a*–*d* presents example acoustic signals. One hundred sixteen German sentences were selected, 29 for each condition, from a larger sample based on ratings and after extensive matching for psycholinguistic criteria as specified below.

All sentences were in active form, they were all in present tense and untypical words and nonliteral usage were avoided. HC and LC sentences were also matched for sentence length and verb conjugations (see **supplementary materials: Stimuli**). Furthermore, HC and LC sentences always included the same main verb and they constrained critical words to be understood always as nouns, as these different grammatical categories may elicit different event-related potentials (ERPs) (Nobre and McCarthy 1995; Pulvermüller et al. 1999). Although the sentences not always had the same subject, HC and LC sentences were matched for the verb conjugation (either 1st or 3rd singular person). The critical nouns were always at the end of the sentences (i.e., separable particle verbs were avoided) and they had been selected on the basis of a cloze test.

Critical words of the sentence stimuli finally used in the EEG experiment were selected on the basis of a cloze probability test performed by 13 German native speakers, who did not take part in the subsequent EEG study. Sentence fragments were randomly presented one by one; participants had to read the sentences and write up to three possible completions.

Critical words were matched for mean word length (average ± SD of letters: animals 6.1 ± 2.6; tools, 6.5 ± 2.3; *t* = −0.7, *P* = 0.5) and word frequency, computed as the number of occurrences of a word form within the dlex Corpus (http://www.dlexdb.de/, animals, 831.1 ± 1045.6; tools, 804.8 ± 727.5; *t* = 0.11, *P* = 0.91). Furthermore, the critical words were all singular and the grammatical feature case was matched between the two contexts (i.e., similar numbers of nouns in nominative, accusative, dative, and genitive case in HC and LC conditions).

The EEG study was conducted in the electrically and acoustically shielded chamber of the Brain Language Laboratory at the Freie Universität Berlin. The EEG and cloze test studies were programmed using E-prime 2.0.8.90 software (Psychology Software Tools, Inc.). The study consisted of one experimental block in which the 116 sentences were randomly presented to the participants. The sentences order was randomized in three separate lists, each EEG participant was randomly assigned to one of these lists. The interval separating the end of the sentence context and the final (expected or unexpected) word onset was 1100 ms (Fig. 1*a*–*d*). This break was necessary to avoid overlap between the neurophysiological responses elicited by the sentence fragments and the SPPs preceding the final critical word (Grisoni et al. 2017; Leon-Cabrera et al. 2019). The interval between the sentences was 2000 ms; the entire EEG recording lasted ∼20 min. All acoustic stimuli were presented binaurally, through high-quality headphones (Ultrasone HFI-450 S-LOGIC™), at a comfortable hearing level. To reduce the possibility of anticipatory activity induced by second-order thinking (e.g., imagery), participants were instructed to ignore the sounds and to focus their attention on a silent movie (“Journey to the edge of the Universe,” National Geographic 2008) free of humans, tools, and animals, which was presented throughout the EEG recording. Participants were monitored through a camera to ensure that they were not moving and were watching the silent movie. Furthermore, participants were presented with three unannounced control questions about specific details of the movie at the end of the EEG recording. All final EEG participants correctly answered at least two of these questions. To reconfirm the status of the high- and low-constrained sentences used as stimuli, another cloze probability test was performed after the EEG recording with all participants (for details, see **supplementary materials: Cloze probability test procedure**).

#### Electrophysiological Recordings and Preprocessing

The EEG was recorded through 128 active electrodes (actiCAP system, BrainProducts), with the following modifications: The reference was moved from the FCz position to the nose tip, and the electrode occupying the I1 position was moved to the empty FCz position (see **supplementary materials: Electrophysiological recordings**). Offline preprocessing followed standard procedure for ERPs analysis (Luck 2014) (for a description of the steps and their order, see **supplementary materials: EEG preprocessing**).

### Data Analysis

#### Cloze Test and Co-occurrence Frequencies

The “critical word” was the word strongly predicted by a HC sentence fragment, which was also used to complete a matched LC fragment. Predictability (or cloze probability) was quantified as the proportion of participants who named the critical word when being presented with the sentence fragment. Furthermore, subjects were asked to rate how sure they were about each sentence completion (“certainty scores”), which were quantified as a score ranging from 0 (uncertain) to 100 (absolutely sure). A 2 × 2 repeated measures ANOVA with the factors context (HC and LC) and word (animals and tools) was performed on both of these scores, predictability and certainty. Furthermore, co-occurrence frequencies were used as an objective corpus-based measure of the likelihood of fragments to be followed by the critical words. To this end, co-occurrence frequencies were computed as follow: first we extracted, from the “Deutsche Referenzkorpus” (German Reference Corpus) of the Institut für Deutsche Sprache (Institute of German Language) in Mannheim, Germany (DeReKo corpus: https://www1.ids-mannheim.de/kl/projekte/korpora/), the absolute frequencies of the critical word (e.g., Adler) appearing after the main verb and noun of the sentence fragments within one paragraph. Then we normalized these absolute frequencies by dividing them by the joint frequencies of the sentence context’s verb and noun appearing together within one paragraph. Since, in this study, we were mostly interested in semantic relationships between the sentence fragments and the critical words, we considered both the singular and plural forms of the critical and contextual nouns along with all of the verbs’ singular present tense conjugations (i.e., first, second, and third; see **supplementary materials: Correlation analysis**).

#### Prestimulus Anticipatory Activity

**Figure 2:**
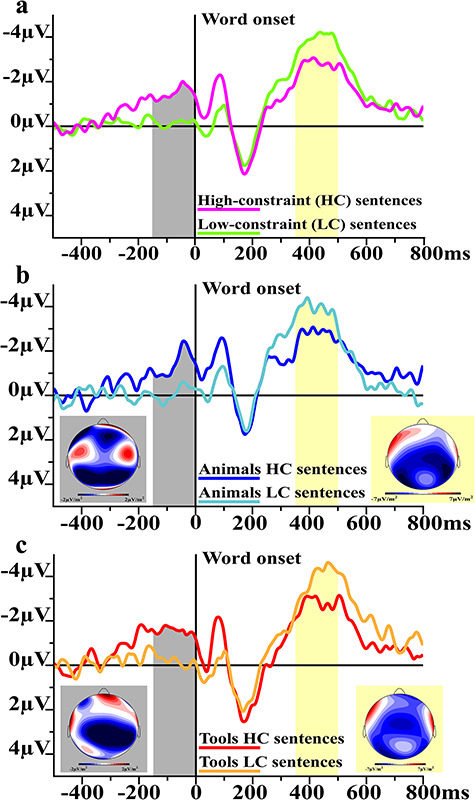
Event-related responses (ERPs). At the top (*a*) electrophysiological responses elicited by HC (magenta) and LC (green) sentences before (SPP time window indicated in gray) and after (N400, in yellow) the onset (time 0) of critical words. SPP before and N400 after animal (blue, central, *b*) and tool words (red, bottom, *c*) are shown for HC and LC conditions. Current source density (CSD) maps for the SPP (gray window) and N400 (yellow window) latencies are reported along with their color scale. CSD maps provide an estimate of the cortical activity after removal of volume-conduction effects.

Any preparatory activity preceding a stimulus must be calculated against a time interval where no or much less such preparatory activity can be expected. Previous work indicates that such anticipatory activity indeed develops a few hundred milliseconds before a predictable critical word appears in semantically highly constrained sentences and is maximal when the expected item appears (Grisoni et al. 2017; Leon-Cabrera et al. 2019). In the present paradigm, spoken sentences were used and the silent interval before the critical word was 1100 ms. To obtain a baseline that included both little event-related activity due to the previous, fragment-final word and, at the same time, a minimum of preparatory activity, we defined the baseline for calculating the preparatory activity between −500 and −300 ms before critical word onset. Figures 2*a*–*c* and 4*a* show that this strategy was successful. More or less predictable contexts did not diverge in their ERP responses during this baseline, whereas there was clear divergence after and a maximal difference just before the critical word appeared.

To determine any ERP differences between HC and LC sentences, a first statistical evaluation was performed using (nonparametric) cluster-based permutation tests, as implemented in the FieldTrip toolbox (Maris and Oostenveld 2007; Sassenhagen and Draschkow 2019). Since predictive neural markers during sentence processing were expected prior the critical words, the cluster permutation test was run on the whole epoch before word presentation (i.e., −300 to 0 ms) on a broad frontoparietal region F1, Fz, F2, FC1, FCz, FC2, C1, Cz, C2, CP1, CPz, CP2, P1, Pz, P2 (Grisoni et al. 2017; Leon-Cabrera et al. 2019). The cluster-based permutation test was computed by randomly exchanging data between the two conditions (i.e., HC and LC) and producing the maximal positive and negative cluster of each permutation (5000 permutations). Clusters were defined as significant only if their occurrence was below *P* = 0.05. Cluster-based permutation test was followed by *t*-test and repeated measures analysis of variances (ANOVA) to test for more fine-grained temporal and topographical extent of the effects. Since any predictive activity before critical word onset implies that the N400 response, as normally calculated, is affected by variation in its baseline, we focused our attention on the last 150 ms before word onset. Indeed, a time window of 100 or 200 ms prior to critical word onset is the most commonly used baseline interval for calculating the N400 (Kutas and Federmeier 2011) (see below). That the cluster-based permutation test revealed the most significant cluster during the last 150 ms before critical word onset (see Results) provided one more reason to focus further investigation on this interval. Finally, previous works indicate that the last 150 ms before word onset include the largest amplitude and greatest signal-to-noise ratio of the anticipatory activity (Grisoni et al. 2017; Leon-Cabrera et al. 2019).

The following analysis steps were taken to assess anticipatory activity: First, the mean amplitude values were calculated for the 150 ms interval at frontocentral electrodes (where this response is typically largest) before critical word onset and the presence of an anticipatory activity was assessed by means of *t*-tests against zero. Second, these mean amplitudes were submitted to a 2 × 2 repeated measures ANOVA with the factors word (animals and tools) and context (HC and LC). Third, topographical differences between animal and tool words were investigated comparing the mean amplitudes from the last 150 ms before critical word onset from a large array of frontoparietal electrodes (FT7, FC3, FC4, FT8; T7, C3, C4, T8; TP7, CP3, CP4, TP8; P7, P3, P4, P8; PO9, O1, O2, PO10). These data were submitted to a four-way repeated measures ANOVA with the following factors: critical word (2 levels: animals and tools), context (2 levels: HC and LC), gradient (anterior–posterior, five levels), and laterality (left–right, four levels).

#### Word-Evoked, N400 Responses

Low-constraint sentences typically elicit larger postword, N400, responses at midline electrodes as compared with high-constraint sentences (Kutas and Federmeier 2011). To test whether these previous observations hold true for the present data set, a first statistical analysis was performed using (nonparametric) cluster-based permutation test at midline electrodes (FCz, Cz, CPz, Pz) from critical word onset to 500 ms. Since the N400 latency is remarkably constant across studies (Kutas and Federmeier 2011), further statistical evaluations focused on the canonical 350–500 ms postword time window. First, the N400 mean amplitudes were averaged at the four midline electrodes (see above) and submitted to a 2 × 2 repeated measures ANOVA with the following factors: word (animals, tools) and context (HC, LC). The same time window (i.e., 350–500 ms) was used to test for N400 topography modulations. However, since it is well known (Kutas and Hillyard 1980) that the N400 is characterized by a posterior, parietally maximal, distribution, we restricted this analysis to centroparietal electrodes including both the CP electrodes, placed above the primary motor area, and occipital electrodes, placed above visual areas (TP7, CP3, CP4, TP8; P7, P3, P4, P8; PO9, O1, O2, PO10). Therefore, a 4-way repeated measures ANOVA with the factors: critical word (animals and tools), context (HC and LC), gradient (central-posterior, three levels), and laterality (left–right, four levels) were performed.

#### Correlation Analysis

In order to test whether there is a systematic relationship between any preparatory activity preceding the critical word, such as the previously reported semantic prediction potential or SPP, and the N400, we performed Pearson correlation analyses. Please note again that the N400 is normally calculated relative to a baseline just before the critical word and, in order to assess a hypothesis about the behavior of the N400, it is necessary to use such a canonical baseline. Furthermore, given preparatory activity is present in some conditions, one may argue that this preparatory activity is not strictly restricted to the precritical word interval, but it could spread from this interval to after critical word onset and overlay the N100 and even N400 responses. Calculating both preparatory and postcritical word responses against the same baseline (before the preparatory wave) and then correlating the two responses would bear the danger that any significant correlation between both measures is entirely due to a lasting prediction potential overlaying N100 and N400. This consideration provides a second reason to calculate the N400 relative to the established baseline before the critical word (100 ms, see above).

However, in order to further investigate whether the canonical N400 baseline, which may contain much anticipatory activity, biased the correlation analysis, a control analysis was carried out on the N100 response. Indeed, if use of the canonical N400 baseline would lead to an erroneous correlation between preparatory and N400 activity, for example, due to component overlap, one would expect the same pattern of results for both N100 and N400. Furthermore, this control calculation is crucial also because it may allow to disentangle whether any potential functional relationship between preparatory and N400 activity is truly semantic. Note that the critical words were spoken items and most of them included 2 syllables, so that their meaning can be recognized not before ca. 300 ms after their onset. Therefore, it is highly unlikely that at N100 latency (i.e., at 80–200 ms), these critical words already produce a semantic brain correlate of their meaning, which could relate to any semantically related preparatory activity before the critical word. Therefore, if there is a semantic prediction potential (SPP) before critical word onset, one would expect a relationship between SPP and N400 but not between SPP and N100.

For the control correlations, we defined the N100 latency from the grand average response obtained by collapsing all critical words (i.e., animals and tools in both HC and LC conditions). Then, the N100 was calculated as the mean amplitude of a 60 ms time window centered at the local maximum observed at frontocentral electrodes (i.e., at 80 ms). We computed Pearson correlations between the N100 and the preparatory activity, defined as the last 60 ms before word onset. Furthermore, in order to exclude the possibility that different time window widths affected the results, we recomputed new correlations (now with 60 ms time windows throughout) between the mean amplitudes extracted during the last 60 ms before critical word onset and the mean amplitudes extracted from the N400 latency (i.e., 395–455). Finally, in order to reconfirm that the functional relationship between the pre- and postword responses was selective for the N400 and did not apply for the N100, we compared the *r* coefficients observed at these two latencies (i.e., N100 and N400) by means of Fisher’s r to z transformation, which permits assessment of statistical significance of any difference between these (the z scores by determining the observed z test statistic). Specifically, we tested whether the significant correlations observed between SPP and N400 were still significant after having subtracted the correlation coefficients observed at N100 latency. In order to test whether the level of subjective expectation predicted both the SPP and N400 responses, we performed Pearson correlation analysis between the “certainty scores” based on the Cloze test (see supplementary materials: Cloze probability test procedure) and both brain responses (i.e., SPP: last 150 ms before critical word presentation and N400: from 350 to 500 ms from critical word onset). Finally, we also tested whether co-occurrence frequencies (see above) predicted the emergence of the anticipatory activity.

#### Source Estimation

Minimum-norm source estimation was applied to ERP topographies following the standard procedure in SPM12 (Litvak and Friston 2008). Minimum-norm estimation makes minimal assumptions about brain generators underlying a surface topography, and it assumes that all source elements can contribute to the recorded data. The model uses a single source covariance component that encodes identically and independently distributed (IID) sources. This SPM12 method provides a minimum energy solution, similar to the method originally proposed (Hämäläinen and Ilmoniemi 1994), which minimizes the total source power (minimal sum of squares of all sources). For regularization, we did not preselect the signal to noise ratio (SNR); the unexplained variance of the solutions reported was about 10%, which represents a realistic estimate in line with previous literature (Miozzo et al. 2015). We note that, in principle, any surface topography cannot uniquely define an underlying generator constellation, and, therefore, the source localization problem is mathematically ill defined (von Helmholtz 1853); therefore, this method, as any other, cannot overcome the nonuniqueness of the inverse problem. Nevertheless, minimum-norm estimation has the advantage to not require a priori information about source generators (Hauk 2004) that could constraint solutions biasing the results. The cortical mesh consisted of 8196 vertices and it was created using the template structural MRI included in SPM12. The EEG and MRI data were coregistered using three electrodes as fiducials: Fpz, TP9, and TP10; for the forward model, we selected the “EEG BEM” as the EEG head model. Therefore, the average responses (i.e., SPP and N400) were inverted using the IID, minimum-norm inversion type (see above). All the activation maps were smoothed using the Gaussian kernel of full-width at half-maximum (FWHM) of 20 mm.

#### Sources of Anticipatory Activity (SPP)

Source estimation was performed for each semantic context (high and low constraint) and critical word type (animals and tools), thus yielding 4 source images for each experimental subject. First, the anticipatory activity including the last 150 ms before critical word onset was compared between the HC and LC conditions. To test whether the two sentence contexts (i.e., HC and LC) induced different patterns of activation, we averaged the source images across critical word types (i.e., animals and tools) for each context (i.e., HC and LC); then, these average images were compared voxel-by-voxel using paired *t*-test. Comparisons were made with correction for multiple comparisons taking into account all voxels of the whole brain, and, in addition, restricting the analysis to left hemispheric voxels only—as language mechanisms can be expected to be left lateralized (in right handers) and, therefore, predominantly manifest in this hemisphere. Second, we focused the analysis on both HC conditions, comparing contexts that strongly predicted animal versus tool words and, therefore, may lead to reliable predictive brain responses (see Results). Slow wave anticipatory components (as, for example, the Readiness Potentials, RP) are characterized by a gradual development and shift of cortical generators, whereby activity typically moves from high association cortex to modality-specific areas (e.g., from prefrontal to motor cortex). To investigate a possible activation of modality preferential areas just before critical word onset, we computed sources for the last 60 ms prestimulus and contrasted HC animal versus tool conditions by means of whole-brain voxelwise paired *t* test. In addition, these sources were also compared using small volume correction in predefined regions of interest (ROIs), applying voxelwise paired *t* tests and FWE correction. As the predictions addressed activity at different hierarchical levels, ROIs were defined in motor and visual areas including both the low (primary) and higher hierarchical levels. To this end, we created a mask image that included primary motor (BA 4), premotor and supplementary motor (BA 6), primary visual (BA 17), secondary visual (BA 18), associative visual (BA 19, BA 7), and inferior temporal (BA 37) areas of the left hemisphere, using the WFU_PickAtlas (Maldjian et al. 2003). These ROIs were then combined in a unique mask image used as an explicit mask.

#### Sources of the N400

Similarly to the analysis of preparatory activity (see above), we first tested whether the two sentence contexts (i.e., HC and LC) also induced a different distribution of the underlying sources. To this end, the N400 sources were extracted from the canonical latency (i.e., from 350 to 500 ms) and collapsed across the two word types by averaging the source images of the two postword responses (i.e., animals and tools) within each context (i.e., HC and LC). Then, we tested the hypothesis of semantic processing facilitation induced by word preactivations. We focused solely on the unequivocal semantic-like effect, neglecting the comparison between the two LC conditions (i.e., animals and tools) whose interpretation would not be trivial due the possibility of further predictive mechanisms at these relatively late latencies. To this end, we contrasted the HC and LC N400 responses for each word category (i.e., animals and tools). As before, we performed an exploratory whole-brain comparison first and then the hypothesis-driven ROI contrasts. Since we intended to test lower visual and motor areas, the hypothesis-driven ROIs were restricted to the primary and adjacent visual areas (i.e., BA 17 and 18) for the paired *t*-tests comparing HC and LC animal word conditions and to the primary motor area (i.e., BA 4) for the HC versus LC tool word conditions (see Fig. 4*e*,*h*).

For both the SPP and N400 whole-brain exploratory contrasts, *P* values were thresholded at *P* < 0.005 (uncorrected), while for the SPP and N400 ROIs analysis, *P* values were thresholded at *P* < 0.05 corrected for multiple comparisons using the FWE procedure; significant clusters had to be at least 20 voxels larger to be considered.

## Results

### Stimulus Ratings

Cloze probability data from participants who did not take part in the EEG experiment confirmed that HC sentences were more predictable than LC fragments, as revealed by the main effect of the two-level factor context (*F*(1,28) = 201.226, *P* < 0.001, ηp^2^ = 0.88). The very same result was also observed with EEG participants (see Fig. 1*e*,*f*). For the latter, repeated measures ANOVAs revealed a main effect of context for both “cloze probability,” that is, the probability with which the critical word was used to complete the sentence fragment: main effect of context (*F*(1,28) = 336.01, *P* < 0.001, ηp^2^ = 0.92) (Fig. 1*e*) and the “certainty scores” indexing how sure participants were about their completion, scores ranged from 0—very uncertain—to 100—entirely sure: main effect of context (*F*(1,28) = 286.93, *P* < 0.001, ηp^2^ = 0.91) (Fig. 1*f*). Finally, the repeated measures ANOVA on distributional co-occurrence frequencies of the critical words in context revealed a main effect of context (*F*(1,28) = 21.05, *P* < 0.001, ηp^2^ = 0.43) due to higher co-occurrence frequencies in HC compared with LC sentences.

### Semantic Prediction Potentials

The two HC conditions, but not LC sentences, elicited a slow negative-going potential before critical word onset (Fig. 2*a*–*c*) whose smoothly growing shape is consistent with previous reports (Grisoni et al. 2016; Grisoni et al. 2017; León-Cabrera et al. 2017; Leon-Cabrera et al. 2019). The cluster-based (nonparametric) permutation tests performed in the time range prior to word onset revealed two highly significant clusters where significant differences between HC and LC conditions were present. Whereas the first cluster was about 100 ms long (i.e., from about 280 to 180 ms before the critical word) (*P* = 0.01), the second cluster covered the last 150 ms before critical word onset (*P* = 0.004), in both cases HC sentences induced larger negativity compare to LC sentences at all channel locations tested. During the last 150 ms before critical word onset, only the two HC conditions (i.e., animals and tools) elicited a reliable anticipatory activity (*t*-test against zero: animals HC: mean amplitude = −1.39 μV, *t*(23) = −3.28, *P* = 0.01 Bonferroni corrected; animals LC: mean amplitude = −0.05 μV, *t*(23) = −0.11, *P* = 0.92, n.s.; tools HC: mean amplitude = −1.48 μV, *t*(23) = −2.78, *P* = 0.04 Bonferroni corrected; tools LC: mean amplitude = −0.22 μV, *t*(23) = −0.48, *P* = 0.63, n.s.). HC sentences elicited larger anticipatory activity compared with LC contexts during the last 150 ms before word onset (main effect of context: *F*(1,23) = 8.87, *P* = 0.007, ηp^2^ = 0.28). Furthermore, the expectation of different word categories (i.e., animals and tools) elicited anticipatory activity with a different topographical distribution as documented by a significant critical word, gradient, and laterality interaction (*F*(12,276) = 2.4, *ε* = 0.41, adjusted *P* = 0.04, ηp^2^ = 0.09). Planned comparisons revealed that animal word expectations elicited larger SPP responses at frontal left as compared with frontal right (Bonferroni corrected *P* = 0.001) and larger ERPs at left and right posterior parieto-occipital electrodes as compared with frontal right (Bonferroni corrected *P* = 0.02 and *P* = 0.0018, respectively) electrodes, whereas the expectation of tool nouns was more clearly reflected at central as compared with posterior electrodes (Bonferroni corrected *P* = 0.02).

### N400

Responses to the critical words consisted of the typical N100 response peaking at about 100 ms, followed by a positive-going response maximal at about 190 ms and a subsequent negative-going deflection resembling the N400 (see Fig. 2*a*–*c*). Postword analyses focused on the latter response because, upon visual inspection of grand average ERPs, the earlier responses did not give evidence of substantial between-condition differences and previous research using spoken sentences has demonstrated the effectiveness of the N400 as a measure for semantic expectancy violations (Kutas and Federmeier 2011). The cluster-based permutation test run after critical word onset (i.e., from voice onset to 500 ms) revealed one significant cluster with larger negativity for LC compared with HC words at 400–500 ms interval (*P* = 0.04). Repeated measures ANOVA on data recorded from canonical sites (i.e., average of FCz, Cz, CPz, Pz) and latency (i.e., 350–500 ms) confirmed a more pronounced N400 responses in LC as compared with HC sentences (main effect of context: *F*(1,23) = 4.7, *P* = 0.04, ηp^2^ = 0.17). The repeated measures ANOVA performed on data from a larger array of centroparietal electrodes revealed significant main effects of gradient (*F*(2,46) = 9.12, *ε* = 0.64, adjusted *P* = 0.003, ηp^2^ = 0.28) and laterality (*F*(3,69) = 24.77, *ε* = 0.65, adjusted *P* < 0.001, ηp^2^ = 0.52) and a gradient by laterality interaction (*F*(6,138) = 4.71, *ε* = 0.61, adjusted *P* = 0.002, ηp^2^ = 0.17). The N400 was modulated in its topographical distribution by both the word type (i.e., animals and tools) and context as revealed by a significant interaction of the factors critical word context and laterality (*F*(3,69) = 3.73, *ε* = 0.73, adjusted *P* = 0. 03, ηp^2^ = 0.14).

### Correlation Analyses

**Figure 3:**
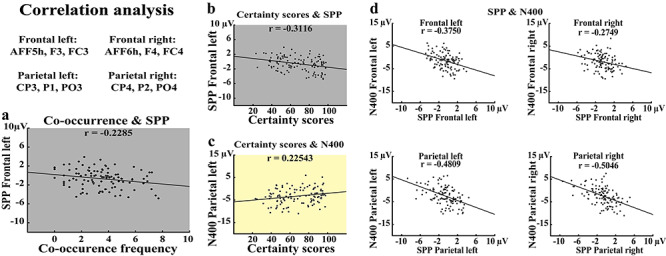
Correlation analyses. Electrodes used for correlation analysis are shown at the top left. All correlations have been computed at sentence level by averaging the electrophysiological responses elicited by all the sentences across all participants. Negative correlation between the co-occurrence frequency and SPP responses at left-frontal electrodes is shown in panel (*a*) (bottom left). Negative correlation between the certainty scores (see Materials and Methods) and SPP responses at left-frontal electrodes is shown in panel (*b*). Positive correlation between the certainty scores and the N400 responses at left-parietal electrodes (i.e., the standard electrodes for N400 responses) is shown in panel (*c*). Negative correlations between SPP and N400 responses at left- and right-frontal and -parietal recording sites are shown, clockwise, in panel (*d*).

*Certainty scores.* Consistent with the SPP frontocentral distribution (Grisoni et al. 2016; Grisoni et al. 2017; León-Cabrera et al. 2017; Leon-Cabrera et al. 2019), the anticipatory activity and the “certainty scores” of the word expectancy showed a significant negative linear relationship, due to the SPP’s negative polarity, at left-frontal electrodes (*r* = −0.31, *P* < 0.002 Bonferroni corrected) (Fig. 3*b*), while the other electrode location showed a weaker, nonsignificant, correlation (frontal-right: *r* = −0.16, *P* = 0.17 Bonferroni corrected). Notably, the N400 and the “certainty scores” showed a significant positive correlation at parietal-left electrodes (*r* = 0.22, *P* = 0.03 Bonferroni corrected) and a nonsignificant trend at the parietal-right electrodes (*r* = 0.18, *P* = 0.09 Bonferroni corrected) (Fig. 3*c*). Thus, whereas the size of the (negative-going) SPP increased with “certainty scores” of the critical word, the (likewise negative-going) N400 shrank.

*Co-occurrence frequency.* To assess whether, similar to certainty scores, statistical regularities in language use as they are manifest in large text corpora are reflected by SPP responses, we tested the linear relationship between the SPP amplitudes with the co-occurrence frequencies of content words in the sentence fragments and the critical, SPP-eliciting words (see section **Data Analysis: Correlation analysis**, and **supplementary materials: Correlation analysis**). We observed a significant negative correlation at frontal-left (*r* = −0.23, *P* = 0.04 Bonferroni corrected) but not at frontal-right (*r* = −0.19, *P* = 0.1 Bonferroni corrected) electrodes. The same analysis performed for all sentences (i.e., including those 14 out of 116 [see **supplementary materials**] where the co-occurrence frequency was 0) yielded similar results (frontal-left: *r* = −0.26, *P* = 0.01 Bonferroni corrected; frontal-right: *r* = −0.16, *P* = 0.15 Bonferroni corrected).

**Figure 4:**
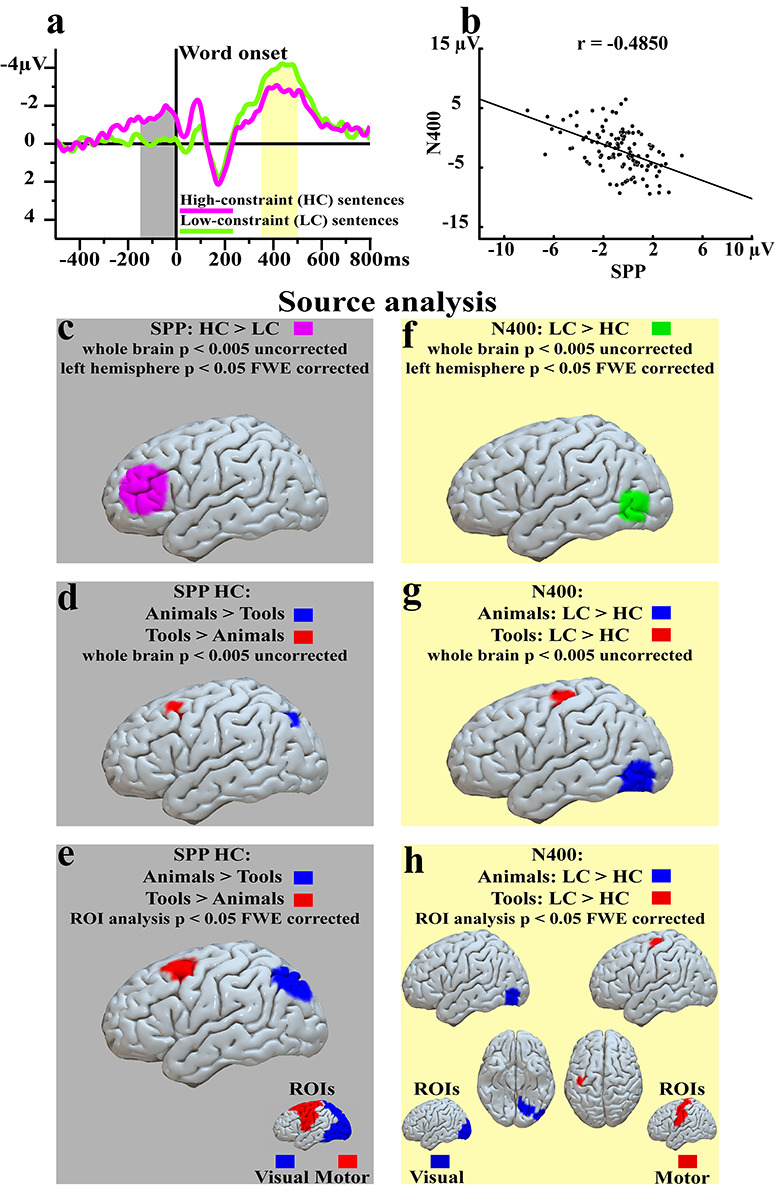
Source analysis results. The top panels summarize main results of this study, SPP (relevant time window indicated by gray shading) and N400 (yellow shading) responses in HC and LC conditions (*a*) and the significant negative correlation between SPP and N400 responses (here averaged across centro-occipital recording sites) in (*b*). Below the top panels, the left column of panels reports sources of SPP contrasts (gray background), whereas the right column gives N400 contrasts (yellow background). (*c*) SPP latency: HC > LC contrast (magenta), *P* < 0.005 uncorrected, whole brain analysis. (*d*) SPP latency: HC animals > HC tools (blue) and HC tools > HC animals (red), *P* < 0.005 uncorrected, whole brain analysis. (*e*) SPP latency, Regions of interest (ROIs) analysis: HC animals > HC tools (blue) and HC tools > HC animals (red) *P* < 0.05 FWE corrected. (*f*) N400 latency: LC > HC contrast, (green), *P* < 0.005 uncorrected, whole brain analysis. (*g*) N400 latency: animals LC > HC (blue) and tools LC > HC (red) *P* < 0.005 uncorrected, whole brain analysis. (*h*) N400 latency, ROIs analysis: animals LC > HC (blue) and tools LC > HC (red) *P* < 0.05 FWE corrected.

*SPP and N400*. The previously reported correlations between SPP, N400, and the measures of contextual predictability (“certainty scores” and co-occurrence in texts) suggest that, as SPP increases, the N400 decreases and vice versa, and thus a negative correlation between the measures of semantic prediction and semantic integration was expected. Consistent with this hypothesis, the SPP (last 150 ms before critical word onset) and the N400 (from 350 to 500 ms after critical word onset) showed significant negative correlations at frontal-left (*r* = −0.37, *P* < 0.001 Bonferroni corrected), frontal-right (*r* = −0.27, *P* = 0.012 Bonferroni corrected), parietal-left (*r* = −0.48, *P* < 0.001 Bonferroni corrected), parietal-right (*r* = −0.50, *P* < 0.001 Bonferroni corrected) (Fig. 3*d*), and centro-occipital (*r* = −0.4850, *P* < 0.001) locations (Fig. 4*b*). The same analyses performed with the shorter time window (i.e., 60 ms) confirmed these significant correlations (frontal left: *r* = −0.42 Bonferroni corrected *P* < 0.001; frontal right: *r* = −0.31 Bonferroni corrected *P* < 0.004; parietal left: *r* = −0.49 Bonferroni corrected *P* < 0.001; parietal right: *r* = −0.52 Bonferroni corrected *P* < 0.001) and at the broad centro-occipital regions (*r* = −0.50, *P* < 0.001).

To test whether these linear relationships reflected semantic processes (or are possibly an artifact of the inclusion of the precritical word interval into the baseline of the N400), we also performed the Pearson correlations between the SPP and the N100 responses which, due to its early latency (i.e., 80 ms), cannot, in the present experiment, index lexical access or semantic integration (for discussion, see Materials and Methods). Consistent with our expectations, the SPP and the N100 did not show significant correlations, neither at the four preselected electrode locations (frontal left: *r* = −0.09 n.s.; frontal right: *r* = +0.03 n.s.; parietal left: *r* = −0.13 n.s.; parietal right: *r* = −0.19 n.s.) nor at the broad centro-occipital regions (*r* = −0.15 n.s.). Finally, we also compared the *r* coefficients observed at N100 and N400 latencies to further test whether the correlations between SPP and N400 were significantly greater than the *r* coefficients obtained between SPP and N100. The results confirmed significantly stronger SPP–N400 correlations at both the four ROIs (two-tailed results: frontal left: Bonferroni corrected *P* = 0.028; frontal right: Bonferroni corrected *P* = 0.036; parietal left: Bonferroni corrected *P* < 0.01; parietal right: Bonferroni corrected *P* = 0.016) and in the broad centro-occipital region (two-tailed result: *P* = 0.003). Overall, these results confirmed a relationship between SPP and N400, but not the N100.

### Source Localization

#### SPP

Consistent with previously reported scalp topographies of anticipatory brain activity (i.e., frontally maximal) (Grisoni et al. 2017; Leon-Cabrera et al. 2019), SPP-eliciting (i.e., HC) sentences induced greater activity in left prefrontal areas, compared with LC sentences (HC > LC whole brain analysis, *P* < 0.005 uncorrected; left hemisphere *P* < 0.05 FWE corrected) (see Fig. 4*c* and Table 1). Exploratory whole brain paired *t*-test comparisons revealed word-related sources in posterior parieto-occipital areas in strong anticipation of animal word presentations as compared with the predictable tool word conditions. The reverse contrast showed relatively greater activation in prefrontal and premotor areas (HC animals > HC tools and HC tools > HC animals, respectively, whole brain *P* < 0.005 uncorrected) (see Fig. 4*d* and Table 1). Hypothesis-driven ROIs in primary and secondary motor and visual areas (see Materials and Methods) confirmed these results (*P* < 0.05, FWE corrected) (see Fig. 4*e* and Table 1).

**Table 1:** Source analysis results

	*x*	*y*	*z*	*t*-values (peak level)	Number of voxels	*P* values	Brodmann areas	Cortical areas
**SPP: Paired *t*-test, whole brain contrast on activation maps obtained by collapsing the two word types: contrast HC > LC**	−48	40	20	3.48	3041	*P* < 0.005 uncorrected	45/46	Inferior left frontal cortex
**SPP: Paired *t*-test, left hemisphere contrast on activation maps obtained by collapsing the two word types: contrast HC > LC**	−48	40	20	3.48	1005	*P* < 0.05 FWE corrected	45/46	Inferior left frontal cortex
**SPP: Paired *t*-test, whole brain contrast: HC animals > tools**	−30	−72	42	2.87	55	*P* < 0.005 uncorrected	7/19	Superior occipital gyrus
**SPP: Paired *t*-test, whole brain contrast: HC tools > animals**	−34	16	54	2.86	22	*P* < 0.005 uncorrected	6	Frontal cortex
**SPP: Paired *t*-test, ROIs contrast: HC animals > tools**	−34	−74	46	3.06	798	*P* < 0.05 FWE corrected	7/19	Superior occipital gyrus
**SPP: Paired *t*-test, ROIs contrast: HC tools > animals**	−36	14	56	3.12	305	*P* < 0.05 FWE corrected	6	Frontal cortex
**N400: Paired *t*-test, whole brain contrast on activation maps obtained by collapsing the two word types: contrast LC > HC**	−48	−72	0	3.13	524	*P* < 0.005 uncorrected	19/37	Occipitotemporal cortex
**N400: Paired *t*-test, left hemisphere contrast on activation maps obtained by collapsing the two word types: contrast LC > HC**	−50	−72	0	3.21	241	*P* < 0.05 FWE corrected	19/37	Occipitotemporal cortex
**N400: Paired *t*-test, whole brain contrast: animals LC > HC**	−36	−70	−8	3.18	1474	*P* < 0.005 uncorrected	18/19	Occipital cortex
**N400: Paired *t*-test, whole brain contrast: tools LC > HC**	−34	−18	62	3.10	103	*P* < 0.005 uncorrected	4	Precentral gyrus
**N400: Paired *t*-test, ROIs contrast: animals LC > HC**	−46	−76	−16	3.22	183	*P* < 0.05 FWE corrected	18/19	Occipital cortex
	−26	−60	2	3.69	296	*P* < 0.05 FWE corrected	18/19	Occipital cortex
**N400: Paired *t*-test, ROIs contrast: tools LC > HC**	−36	−18	62	3.44	46	*P* < 0.05 FWE corrected	4	Precentral gyrus

For all significant contrasts calculated on the cortical sources of the SPP and N400 time intervals, the table displays the MNI coordinates of the voxel with highest *t* value, its *t* value, the number of significant voxels per each significant cluster, *P* values, and the Brodmann area labels where the “peak voxel” was found, along with a description of the cortical area where the active cluster was located.

#### N400

Consistent with the N400’s well-known posterior distribution (Kutas and Federmeier 2011), N400-eliciting LC sentences showed greater activations in temporo-occipital areas compared with HC sentences at the canonical N400 latency (i.e., 350–500 ms) (LC > HC whole brain analysis, *P* < 0.005 uncorrected; left hemisphere *P* < 0.05 FWE corrected) (see Fig. 4*f* and Table 1). Dependent on the semantic content of the unexpected critical words, sources were relatively more prominent in posterior visual areas, in proximity of primary visual area (V1), for animal words (animals LC > animals HC) but in the motor system, in proximity of primary motor cortex (M1), for tool words (tools LC > tools HC) (whole brain analysis, *P* < 0.005 uncorrected) (see Fig. 4*g* and Table 1). The same contrasts carried out in hypothesis-driven ROIs (see Materials and Methods) confirmed these results (*P* < 0.05 FWE corrected) (see Fig. 4*h* and Table 1).

## Discussion

Sentence contexts constraining the expectation of critical words with visually (i.e., animals) or action- and visually related (i.e., tools) semantic meaning induced larger semantic prediction potentials (SPP) (Grisoni et al. 2019; Pulvermüller and Grisoni 2020) before critical word presentation, as compared with low-constraint sentences (see Fig. 2*a*–*c*). That the SPP is genuinely related to semantics was shown by significant correlations between its amplitude with both subjective reports of the words’ predictability in their semantic context (“certainty scores”) and objective corpus-based measures (i.e., co-occurrence frequencies of the critical words in context, see Fig. 3*a*,*b*). The same critical words presented in low-constraint sentence contexts elicited substantially larger postword N400 responses compared with their presentation in high-constraint contexts, thus confirming a well-known observation in N400 research (Kutas and Federmeier 2011) (see Figs 2*a*–*c* and 4*a*). One crucial expectation was to observe a correlative relationship between activations observed before and after the critical words. Notably, the N400 linearly decreased with larger anticipatory activity as documented by the significant negative correlations between these two brain signatures (see Fig. 3*d* and 4*b*), thus suggesting a functional relationship between pre- and postword brain activations (see Discussion below). The correlation between SPP and N400 was specific, as correlations between the SPP and the N100 were not statistically significant, thus arguing against the possibility that the observed correlations of the N400 might have been due to the canonical preword baseline, which contained some of the anticipatory activity. Furthermore, the difference test on the correlation coefficients confirmed that the correlations observed between SPP and N400 was significantly greater than that between SPP and N100. These results raise the possibility that the specific correlation between the brain indexes of anticipation and integration of the critical words may result from a causal relationship between semantic prediction and subsequent N400-relevant processes such as prediction error computation, verbal memory access, and/or context integration (see also section on SPP-N400 relationship).

Consistent with previous reports (Lau et al. 2008; Alexander and Brown 2018), the main brain generators underlying the SPP and the N400 responses were located in inferior prefrontal cortex (Fig. 4*c*) and in posterior temporal cortex (Fig. 4*f*), respectively. Furthermore, similar to previous reports about word-evoked potentials following the critical item at different latencies (Kiefer 2001; Carota et al. 2012; Carota et al. 2017), the expectation of animal nouns produced additional anticipatory activation in posterior visually cortical areas, possibly due to the processing of visually related object knowledge about their referents; in contrast, tool noun expectations were reflected by additional prefrontal and motor area activity, which may in part reflect knowledge about the action-related function and use of tools (see Fig. 4*d*,*e*). Consistently, the sources underlying the enlarged N400 responses to not-predicted animal and tool words lay adjacent to the same modality preferential brain areas, in higher visual- and action-related areas, respectively (see Fig. 4*g*,*h*). Overall, these results show that the SPP has similar semantic discriminatory function as the N400.

### Function and Cortical Sources of the Semantic Prediction Potential

Previous research established that predictable stimuli elicit slow anticipatory activity before they appear (Kilner et al. 2004; Grisoni et al. 2016; Grisoni et al. 2019) and, consistently, sentence fragments constraining the expectation of specific subsequent words give rise to similar responses with prominent frontocentral distribution (Grisoni et al. 2017; Leon-Cabrera et al. 2019) (Fig. 2*a*–*c*). Event-related potentials (ERPs) are traditionally classified taking into consideration the cognitive process they index, hence the choice of the term “semantic prediction” potential, SPP. The data here presented show that semantic predictability is the psychological variable reflected by the SPP: high- but not low-constraint sentence fragments gave rise to anticipatory waves (Fig. 2*a*–*c*), and there was a correlation between ratings in the cloze test and the magnitude of the SPP (Fig. 3*b*). The cloze test is an established estimate of semantic expectancy also applied in many N400 studies, and it has previously been shown that the N400 is inversely related to this variable (Kutas and Hillyard 1984). In addition, the SPP’s amplitude correlated with an objective corpus-based measure of semantic predictability, the co-occurrence frequency of the critical word in the context of key content words in the sentence fragment (Fig. 3*a*). Whereas the former observation provides evidence from a psycholinguistic perspective, the latter draws on distributional semantic information and suggests a role of statistical regularities in establishing semantic memory representations with predictive properties (Keysers and Gazzola 2014). After seeing a cow, for example, we may not be surprised to hear its typical sound and a sentence about it will likely mention related features or things, such as a cow’s properties, its typical activities, or the names of other farm animals, and it is generally accepted that the brain is able to internalize such statistical regularities relevant to distributional semantics (Kuhl 2004; Pulvermüller 2018). Therefore, the correlative link between word co-occurrence frequency and SPP would suggests that predictive coding might emerge as a consequence of associative learning of word co-occurrences (Pulvermüller 2018). It is possible that a word is anticipated in a given context based on the listener’s previous experiences of occurrences of the word in that context, although other possible learning mechanisms including generalization should also be considered.

As mentioned, we already documented a semantic prediction potential in a previous study also using a paradigm with sentence fragments constraining target words (Grisoni et al. 2017). In that work, sentence fragments either predicted specific action verbs or, similar to the typical N400 experiment, these same predictions were crudely violated. It can be criticized that these data do not allow for deciding whether the predictions made by the neurocognitive system are word specific or rather address a semantic group of semantically very similar words (Pickering and Gambi 2018). Therefore, in the present study, we chose sentences in such a way that the predictions induced by the fragments, for example, “The emblem of Germany is the …,” were rather specific, only allowing one critical word, “eagle” (note that semantically close items such as “hawk,” “buzzard,” or “vulture” would constitute clear expectance violations). Therefore, we believe that the above criticism cannot be brought up against the current results: Predictions and prediction errors were item specific. Still, our present study focused on contexts with narrowly predictable and unpredictable critical words, with intermediate cases still calling for further research. Furthermore, by contrasting fragments strongly and weakly predictive of specific words, we cannot address whether predictions at different linguistic levels (of whole semantic categories, syntactic classes, or phonological features) can be equally manifest in predictive brain activity. The absence of any SPP in the present low-constraint condition suggests that a very unspecific prediction of, for example, any member of a broad semantic class does not become manifest as an SPP. Whether this is an indication of absence of prediction remains to be investigated. Our present data broaden the previously reported results (including Grisoni et al. 2017) by showing that not only predictions on action-related verbs but also those on nouns from variable and rich semantic categories, that is, animal and tool nouns, are cortically manifest as specific anticipatory activity with well-defined cortical origin.

A further key issue assesses the SPP’s generators in the brain. Consistent with the frontal distribution of the SPP (Grisoni et al. 2017; Leon-Cabrera et al. 2019), we here showed that its most prominent source is located in inferior prefrontal cortex (see Fig. 4*c*). Overall, this result is consistent with previous reports showing an involvement of lateral prefrontal areas in anticipating predictable stimuli (Grisoni et al. 2019). Further converging evidence comes from intracranial cortical recordings in human surgical patients revealing negative slow wave responses in anticipation of repetitive, hence predictable, acoustic stimuli (i.e., tones) in lateral prefrontal cortex (Durschmid et al. 2019). The SPP’s prefrontal distribution is also consistent with the most well-known slow negative potential emerging before motor movement (e.g., button presses), the readiness potential (RP) (Deecke et al. 1969), which typically originates from prefrontal territories (Haggard 2008; Kappenman and Luck 2012).

Whereas the RP’s sources are confined to the frontal lobes, SPP sources changed significantly depending on what kind of meaning was expected. Our previous observations had indicated this already (Grisoni et al. 2017), although they had been limited to sentences in which the predictable critical words referred to body part–specific actions, in particular face (e.g., “lick”) and hand-related (e.g., “write”) action verbs. Therefore, these previous results still left it open to a degree whether the observed SPP sources in frontal and motor cortices were due to specific semantic features of the expected critical words. Our results now show that the frontal sources are also present for words, which are not used to speak about actions, but to objects instead, and even to objects not related to action (animal nouns). As in the previous study, the present topographical distributions of and sources underlying the SPP were significantly modulated by the semantic type of predicted critical words (Fig. 4*d*,*e*). Contexts predicting animal words elicited additional parieto-occipital activations before critical word presentation (Fig. 4*d*,*e*), possibly due to visual object knowledge about their referents, whereas tool noun expectations became manifest as premotor area activation, which may in part reflect knowledge about the use of tools. These results are consistent with previous reports on the brain correlates of animal and tool words following unexpected critical words (Kiefer 2001; Carota et al. 2012; Carota et al. 2017), which can be interpreted as correlates of the understanding and context integration of aspects of the referential semantic meaning of these symbols (Martin 2007; Pulvermüller 2018).

Our present results show that local dissociations in neurometabolic activation similar to those observed as correlates of the meaning of words in response to these items can appear before these same words appear in contexts strongly predict them. The four bottom panels of Figure 4 illustrate the similarity of these dissociations: Relatively stronger motor/premotor/prefrontal area activations are present for tool words, but relatively stronger parietal/occipital visual area activations for animal words, as revealed by both SPP and N400. It can be seen that these prediction-related (SPP sources) and the comprehension- and context integration–related (N400 sources) brain responses are in the same cortical systems (action vs visual) for both semantic types, although there are local differences. This correspondence is consistent with the position that semantic dissociations are similarly manifest in cortex during prediction and understanding of a meaningful symbol. (We discuss possible reasons for the activation differences below.)

Considering the similarities between SPP and RP, in terms of topographies and main sources, one may suggest that these components are related to each other, although the SPP has a much broader scope than the RP. Whereas the RP is always seen before overt action, with sources restricted to frontocentral cortex “predicting” the body part with which a movement will be carried out, the SPP indexes expectations of both action- and perception-related information and, correspondingly, includes additional cortical generators in motor or sensory (auditory or visual) areas, dependent on the nature of the predicted items. Note again that, apart from semantic word-related expectations, previous studies have reported similar anticipatory activity before predictable nonaction-related sounds (e.g., tones, water drops) (Durschmid et al. 2019; Grisoni et al. 2019), action-related visual stimuli (Kilner et al. 2004), and action sounds (i.e., whistle, hand clap, and footstep) (Grisoni et al. 2016; Grisoni et al. 2019). These anticipatory responses showed predictive sources located within the relevant modality preferential (e.g., motor, auditory) brain areas, thus suggesting that the SPP is a brain signature of the preactivation of specific memory circuits storing information about the expected stimulus.

### The Relationship between SPP and N400 Responses

Here, we also investigated postword responses, in particular N100 and N400, and their relationships to predictive brain activity. It is well known that N400 amplitudes are larger for semantically unpredictable than to predictable sentences (Kutas and Hillyard 1984) and, consistently, we here report that SPP-eliciting predictive (HC) sentence fragments showed weaker N400s to the critical words as compared with unpredictive (LC) ones, which, in turn, did not show any reliable SPP before the critical word (Fig. 2*a*–*c*). Furthermore, the correlation between subjective reports of word expectancy, quantified as the certainty with which critical words could be predicted and as co-occurrence probability in texts, and N400 amplitudes confirmed that expectancy was systematically related to N400 amplitude (Fig. 3*c*). Pickering has, recently, stressed how difficult it is to determine with certainty whether brain indexes such as the N400 uniquely index prediction and the resultant preactivation of lexicosemantic items, or rather other processes related to the integration of a word in a context. Note that, rather than prediction and preactivation of a given word, or group of words, it could also be that N400 size indicates the ease or difficulty in matching a (preactivated or not-preactivated) lexical item with its context, the shared semantic features between context and target or other psycholinguistic features, which also may relate to predictability (Pickering and Gambi 2018). Indeed, some colleagues have argued that the enlarged N400 to unpredictable words would not index prediction error but, rather, the processing load required to integrate the critical word in its semantic context and, notably, this interpretation is quite well established in the literature (Brown and Hagoort 1993; Kutas and Federmeier 2011). To show that the N400 is a possible prediction error signal, it is of utmost importance to have available a brain signature of prediction emerging before word presentation and, therefore, independent of integration. Therefore, the negative correlations between the N400 and the SPP responses here reported—but not between the prelexical N100 and the SPP—(Figs 3*d* and 4*b*) are important to establish the possible role of the N400 as a genuine prediction error signal. In this respect, the N400 resembles other neurophysiological measures, most notably the mismatch negativity, or MMN, (Näätänen et al. 2001), for which we reported in a previous study linear relationships with predictive brain activity (Grisoni et al. 2019). However, in contrast to the MMN, which may indicate prediction errors at different perceptual and cognitive levels (Garrido et al. 2008; Grisoni et al. 2019), the N400 specifically indicates failures in predicting symbols at the semantic level.

This study compared high-constraint, HC, conditions, in which a strongly predicted word appears in sentence-final position, to low-constraint, LC, conditions, which lacks any strong prediction on sentence final words and, consequently, the final target word is to a degree unexpected. For sentences of these types, which are common in everyday language use, we found a negative correlation between predictive and N400 responses. This observation is consistent with a theoretical proposal according to which linguistic predictions are cortically manifest as preactivation of the neuronal circuits for words or groups of lexical items. The more a circuit is preactivated, the less additional activation will be needed to fully activate this same circuit in response to the occurrence of the critical word. This putative mechanism provides a possible explanation of the negative correlation between SPP—the presumed index of preactivation—and the N400—the established indicator of (difficulties in) context integration and/or verbal memory access. This model further suggests that the cortical mechanisms reflected by SPP and N400 (and other prediction-error indexes such as the MMN) are causally related to each other: The preactivation of the lexical trace facilitates its full ignition and further processing in the language network. However, we note that, what we observed and report are correlations between brain responses. Any firm conclusions on causality must, therefore, remain tentative until further evidence is available.

One may argue that the observed correlation between SPP and N400 may depend on the selection of conditions in the present experiment and that the inclusion of further conditions may remove (or substantially weaken) this correlation. A possibility already mentioned in the Introduction would have been to present typical “N400 violations” with critical words violating strong semantic expectations raised by HC sentence fragments (e.g., “The emblem of Germany is the … vulture”). For these items, strong semantic predictions come with maximal unexpectedness of the critical word. According to the current results, this would imply large SPPs along with large N400s, thus working against a significant negative correlation of these measures. Such a condition had been included in one of our previous studies (Grisoni et al. 2017), where the N400 indeed varied with the predictability of target words, although equally large predictive activity was seen across HC conditions. Therefore, the negative correlation reported here depends on the absence, or rareness, of HC fragments followed by notpredicted items. However, as this type of event is, by definition, very rare in real life (as it has low probability and thus is unlikely to occur), it may not be able to reduce regression coefficients substantially. In an N400 experiment including only HC contexts half of which are followed by very high and low-cloze target words, respectively, no correlation between SPP and N400 is expected. Note however, that such lack of correlation is once again consistent with the causal model according to which, in the N400 violation condition, the predictable item(s) would be strongly preactivated (thus yielding a large SPP) and an unprimed, not-preactivated circuit would be ignited by the low-cloze critical word (resulting in a large N400).

Given the correlative relationship between SPP and N400 in the present experiment, it is now necessary to consider whether the sources underlying the pre- and postword responses are consistent with a partly shared mechanism. In partial agreement with the typical posterior distribution (Kutas and Federmeier 2011) and with previous reports on N400 source estimations (Helenius et al. 1998; Lau et al. 2008), the main N400 generator was located in posterior temporal and anterior occipital cortex (Fig. 4*f*), one of the areas traditionally associated with semantic memory access and processing (Hickok and Poeppel 2007; Lau et al. 2008; Price 2012; Pulvermüller 2013). In contrast, the main source of the SPP was found in prefrontal cortex, thus suggesting that processing in substantially different brain regions supports the generation of predictions and prediction error processing. However, we found additional sources dependent on semantic word categories. The enlarged N400 responses to unpredictable as compared with predictable sentences lay within modality preferential brain areas, with unpredicted animal nouns showing additional activity in posterior ventral temporal areas as compared with predictable nouns (Fig. 4*g*,*h*), and unpredictable as compared with predictable tool nouns originating in part in or close to motor areas (Fig. 4*g*,*h*). Crucially, as pointed out above, these results about the sources of semantic word category differences are similar to those observed at preword (SPP) latency (Fig. 4*d*,*e*), thus confirming a partial resemblance between these two brain responses (see also Discussion below). Overall, these results indicate that the N400 has an antecedent, the SPP, with similar semantic discriminatory function and sources. We, therefore, suggest that overlapping or closely adjacent semantic memory circuits were involved in predictive and integration-related activations.

### Hierarchical Predictive Coding

Although pre- and postword source estimations revealed clusters of activity within the same modality preferential brain areas (i.e., the wider visual and motor systems), their exact localization was different (Fig. 4*d*–*h*). Indeed, the pre- and postword sources differed in relation to the level of the cortical hierarchy, in which activations specific to semantic types (i.e., symbols referring to animals vs tools) emerged. Whereas semantic expectations were indexed by relatively greater activation further away from primary cortices (Fuster 2004), in dorsal-parietal visual and posterior-prefrontal areas, postword processing of previously unexpected words differentially activated the corresponding modality preferential territories close to, or overlapping with, primary fields and therefore at “lower” hierarchical levels (Fig. 4*d*–*h*). Although this pattern of results is difficult to explain and may in part be due to the different control conditions used for subtraction (see the 4 bottom panels of Fig. 4), this pattern of results is consistent with hierarchical predictive coding, a neurobiologically informed theory of brain function (Felleman and Van Essen 1991; Rao and Ballard 1999; Friston 2010; Bastos et al. 2012). According to this framework, each level in the hierarchical network attempts to predict the activity at lower levels and, therefore, in “lower areas” (Huang and Rao 2011), in order to aid the suppression of any ascending neuronal activity that could be anticipated. Whereas some PC accounts focus on the columnar organization of cortical layers and attribute relatively higher hierarchical levels of processing to deeper layers (Friston 2010), other accounts (Rao and Ballard 1999) suggest a functional hierarchal organization across cortical areas (Felleman and Van Essen 1991; Badre and D'Esposito 2009). Our data can obviously not speak to the layer level but appear to be consistent with increasingly higher levels of processing when moving away from primary toward multimodal association cortex. For example, it has been suggested that auditory prediction error responses, such as the mismatch negativity or MMN, emerge from a feedforward–feedback cascade, in which higher-order frontal areas generate top-down predictions reaching lower perceptual areas (including superior temporal gyrus), where prediction error signals are computed based on the match or mismatch between top-down prediction and the stimulus information reaching primary auditory cortex (Garrido, Kilner, Kiebel, et al. 2009). Recent results indicate that the classical MMN prediction error signal originates from a hierarchy of predictive coding mechanisms, both in nonhuman mammals (i.e., rats and mice) (Parras et al. 2017) and humans (Dürschmid et al. 2016). Consistent with this, Parras and colleagues (Parras et al. 2017) reported largest prediction error signals in superior temporal lobe, close to auditory cortex. Overall, our results are consistent with this theoretical approach and recent experimental evidences, as prediction potentials emerged in areas further away from sensorimotor fields as compared with poststimulus error signals, which emerged in and close to primary areas.

In conclusion, our results show physiological correlates of the interplay between semantic prediction and integration processing in sentence understanding. These correlates of prediction and integration were manifest in signal and source space. In signal space, larger preword *predictive* responses, that is, SPPs, were followed by smaller postword N400 activations, which can be interpreted as prediction error related, because there is now physiological evidence for the presence of predictions revealed by the SPP. Vice versa, the absence of a (strong and reliable) prediction, as signified by the absence of the SPP, results in a large N400, now interpretable as an error signal. These results reveal a putative neurobiological correlate of the interplay between predictive coding and prediction resolution in language understanding. In source space, the main generators underlying prediction and resolution were markedly different, lying, respectively, in prefrontal and posterior temporal cortex, but there were additional sources indicated semantic differences between semantic word categories, and these differential sources were similar and adjacent across prediction and resolution. In showing the interplay between brain correlates of prediction and prediction error and resolution in semantic understanding, the present results may be of relevance to future work in linguistics, neuroscience, and cognitive science.

## Supplementary Material

Supplementary_Materials_Grisoni_et_al_bhaa308
