# Supplementary material for: Correlated Brain Indexes of Semantic Prediction and Prediction Error: Brain Localization and Category Specificity
Source: Cereb Cortex. 2020 Oct 27;31(3):1553–68. doi: 10.1093/cercor/bhaa308 (PMC7869099; doi:10.1093/cercor/bhaa308)
Supplement: Supplementary_Materials_Grisoni_et_al_bhaa308 [file supplementary_materials_grisoni_et_al_bhaa308.docx]

**Correlated brain indexes of semantic prediction and prediction error: brain localization and category specificity**

**Luigi Grisoni^1*^, Rosario Tomasello^1,2,3^ & Friedemann Pulvermüller^1,2,3,4^**

**Affiliations:**

*^1^ Freie Universität Berlin, Brain Language Laboratory, Department of Philosophy and Humanities Habelschwerdter Allee 45, 14195 Berlin, Germany.*

^2^ *Berlin School of Mind and Brain, Humboldt Universität zu Berlin Luisenstraße 56, 10117 Berlin, Germany.*

*^3^ Cluster of Excellence ‘Matters of Activity. Image Space Material’, Humboldt Universität zu Berlin Unter den Linden 6, 10099 Berlin, Germany.*

*4 Einstein Center for Neurosciences Charitéplatz 1, 10117 Berlin, Germany*

*Correspondence to:

Luigi Grisoni, Ph.D.

Brain and Language Laboratory

Department of Philosophy and Humanities

WE4 Freie Universität Berlin,

Habelschwerdter Allee 45, 14195 Berlin, Germany.

Phone: +49 (0)30 838-58783,

e-mail: grisoniluigi@zedat.fu-berlin.de

Keywords: semantic processing, Predictive coding, Semantic Prediction Potential (SPP), N400

**Supplementary materials**

**Stimuli**

| ***Sentences’ length*** | ***Number of words*** | ***SD*** |
| --- | --- | --- |
| ***Tools HC*** | 7.07 | 1.39 |
| ***Tools LC*** | 6.66 | 1.14 |
| ***Animal HC*** | 6.72 | 1.06 |
| ***Animal LC*** | 6.76 | 1.06 |

**Cloze probability test procedure.**

After the EEG recording, participants were seated in front of a PC and they were asked to evaluate the sentences in a cloze probability test. Participants were instructed as follows: “You will hear several incomplete sentences. Please write down which words you would use to complete each sentence you hear along with a number from 0 to 100, this number has to express how sure you are about your completion (0 very unsure, 100 very sure *certainty scores*). You can write one, two, or three possible completions with one or two words. If you do not have any idea, please don't write anything down.” Therefore, the participants had to listen to sentence fragments (i.e., the stimuli sentences without the critical word) and write down the words they would expect in the respective contexts. Upon responding, they were presented with the next incomplete sentence. The sentence order was randomized for each participant.

**Electrophysiological recordings.** The EOG signals were recorded with one electrode placed below the left eye and with two electrodes embedded in the fabric cap that were placed on the side of each eye (i.e. F9 and F10). During data acquisition, the EOG channels had the same reference as all the other EEG electrodes. All electrodes impedances were kept below 10 kΩ. Data were amplified and recorded using BrainVision Recorder software (version: 1.20.0003; Brain Products GmbH, Gilching, Germany) with a pass-band of 0.1-250 Hz and a sampling rate of 1000 Hz. Data were stored on a disk.

**EEG pre-processing.** Offline analysis started with a digital high-pass filter (Butterworth zero phase filter, 0.1 Hz low cutoff, 12 dB/oct) that, to avoid edge artifacts, was applied on the raw, unsegmented data. Therefore, raw data have been epoched in large segments, from 600 ms before word onset to 2000 ms after. Afterwards, independent component analysis (ICA) based on the default infomax algorithm ‘runica’ (Bell and Sejnowski 1995), as implemented in EEGLAB 13 (Swartz Center for Computational Neuroscience, <http://www.sccn.ucsd.edu/eeglab>), was carried out on all the 128 electrodes. A component was classified as artifactual, when its topography showed peak activity only over the horizontal or vertical eye electrodes and when it showed a smoothly decreasing power spectrum, which is typical for eye movements (Delorme and Makeig 2004). On average, 3 out of 128 components were removed from each participant’s dataset. After having classified the artifactual ICA-components, the EEG signal was corrected by removing the eye artifact components from EEG data, using the standard function implemented in EEGLAB 13. After artifact correction, off-line analysis was performed with BrainVision Analyzer (Brain Products GmbH, Munich, Germany). First, bipolar EOG channels were created with the intent to better isolate residual post-ICA artifact. To this end, the new VEOG channel was obtained subtracting the Fp1 from the lower eye electrode, while the new HEOG channel by subtracting the right (i.e. F10) from the left (i.e. F9) eye electrode signal. The EEG signal was then low-pass filtered with the Butterworth zero phase filter, 20 Hz and notch filter 50 Hz (24 dB/oct), which are typical filter settings for both the N400 and the slow brain potentials (Kappenman and Luck 2012). Trials were shortened from 500 ms before word onset to 800 ms after. The first 200 ms of the segmentation were used as baseline. Epochs with voltage fluctuation of > 100 μV at any electrode or with voltage fluctuation of > 80 μV at EOG channels, and those contaminated with artifacts due to amplifier clipping, burst of electromyographic activity, or alpha power were excluded from averaging, amounting to approximately 16.9% of all trials (range 6 – 27.6%).

**Correlation analysis: co-occurrence**. For the correlation analysis the co-occurrence frequencies were transformed logarithmically. However, since in 14 sentences, out of 116, the co-occurrence frequencies were zero, we performed the correlations twice. In one case, we changed all the zeros with 0.001 (i.e. ln = -6,90775), whereas in a second run we removed them.

**Correlation analysis: Regions of Interest (ROIs).** All the correlation analyses were performed on the values obtained by averaging event-related EEG activity across all participants’ data for each sentence, thus resulting in 116 data points for each of the two components. Since these two waveforms tend to have a slightly different topographical distribution (SPP anterior-prefrontal, N400 posterior-parietal) (Kutas and Federmeier 2011; Grisoni et al. 2017), we considered two SPP-frontal regions of interest (ROIs), and two N400-posterior ROIs. These ROIs were defined as the average of three neighboring electrodes (SPP ROI: Frontal-left: AFF5h, F3, FC3; SPP ROI: Frontal-right: AFF6h, F4, FC4; N400 ROI: Parietal-left: CP3, P1, PO3; N400 ROI: Parietal-right: CP4, P2, PO4) (**Figure 3**). Correlations involving the SPP and the N400 alone (i.e. with the certainty scores and the co-occurrence frequencies) were computed on their respective ROIs (i.e. SPP-frontal and N400-posterior). Vice versa, the correlation analysis between the two components (i.e. SPP and N400) was computed both on all the four ROIs and on a larger centro-occipital region (i.e. average of C5, C3, C1, Cz, C2, C4, C6, CP5, CP3, CP1, CPz, CP2, CP4, CP6, P5, P3, P1, Pz, P2, P4, P6, PO7, PO3, POz, PO4, PO8, PO9, O1, Oz, O2, PO10). All the correlation results were corrected for multiple comparisons using Bonferroni correction.

**References**

Bell AJ, Sejnowski TJ. 1995. An information-maximization approach to blind separation and blind deconvolution. Neural Comput 7:1129-1159.

Delorme A, Makeig S. 2004. EEGLAB: an open source toolbox for analysis of single-trial EEG dynamics including independent component analysis. Journal of neuroscience methods 134:9-21.

Grisoni L, Miller TM, Pulvermüller F. 2017. Neural correlates of semantic prediction and resolution in sentence processing. The Journal of neuroscience : the official journal of the Society for Neuroscience.

Kappenman ES, Luck SJ. 2012. The Oxford Handbook of Event-Related Potential Components. In: 'Oxford University Press'.

Kutas M, Federmeier KD. 2011. Thirty years and counting: finding meaning in the N400 component of the event-related brain potential (ERP). Annual review of psychology 62:621-647.
